# Editorial: Women in interacting minds and brains II

**DOI:** 10.3389/fnhum.2026.2001078

**Published:** 2026-10-26

**Authors:** Nayara Mota, Anna Ilona Roberts

**Affiliations:** 1 Institute of Psychology and Social Work, Federal University of Bahia, Salvador, Bahia, Brazil; 2 Catholic University of Salvador, Salvador, Bahia, Brazil; 3 Institute of Psychology, University of Wrocław, Wrocław, Poland; 4 Bonobo Foundation, Chester, United Kingdom

**Keywords:** gender, neurocognition, neurophysiology, social neuroscience, women

Women's participation in strategic and leadership positions in neuroscience remains disproportionate to their participation at earlier career stages, reflecting persistent limitations in career progression. This disparity was already documented by the Society for Neuroscience 14 years ago: women represented 56% of students entering graduate training in neuroscience, but only 29% of tenure-track faculty and 23% of full professors, with little change in senior representation since 2000 (Society for Neuroscience, 2012). Contemporary evidence suggests that this gradient persists. From 2002 to 2020, women accounted for 36.2% of first authors but only 24.6% of last authors in PubMed-indexed neurological publications (Nguyen et al., 2021). Among neurology researchers, women were also less likely to be full professors (18% vs. 39% men), reported greater nonprofessional responsibilities and lower work–life balance satisfaction, and perceived less equitable institutional climates (Hall et al., 2023). Qualitative evidence further identifies barriers including limited access to women mentors, under-representation in senior positions, competing family responsibilities, disparities in compensation and promotion, and experiences of bias and harassment (Dy-Hollins et al., 2024). Together, these findings indicate that gender disparities extend beyond entry into neuroscience to sustained progression toward scientific leadership.

By focusing on research led by women, this Research Topic contributes to changing the current neuroscientific landscape. Beyond reducing gender inequality in science, the greater representation of women in neuroscientific research may broaden theoretical and methodological approaches and contribute to reducing potential biases in the research questions, objects of study, and interpretations of neuroscientific evidence. Differences in neurocognitive and neurophysiological processing between women and men, highlighted by some contributions to this Research Topic, may also be embedded in social and cultural differences. Greater diversity among researchers may broaden perspectives in neuroscience and counterbalance biases arising from predominantly male authorship.

Thus, this Research Topic contributes in two complementary directions: presenting scientific contributions led by women and highlighting sex- and gender-related differences in neurocognitive and neurophysiological processes, mediated or not by social aspects. Regarding the first, research led by women in this Research Topic places particular emphasis on the social dimensions of human neuroscience. In this sense, empathy was incorporated into the analysis of individual differences in brain responses to robot and human pain (Chin et al.). Advancing human-robot interaction research, the authors found prefrontal cortical hemodynamic responses to robot pain equivalent to the ones to human pain. Despite the absence of differences in the measures of near-infrared spectroscopy (fNIRS), participants were able to verbally discriminate robot pain faces from neutral ones, as well as from human pain faces. In another study from this Research Topic, social bondings and social identity were highlighted as important dynamics developed through food culture among primates, as they are in human groups (Roberts and Roberts). Based on an integrated theoretical model of dietary traditions and primate social complexity, this work provides an evolutionary perspective on how culture integrates biological and social demands.

Considering both contributions from this Research Topic, three studies collectively examine sex- and gender-related differences across complementary levels of neurocognitive and neurophysiological analysis: behavioral testing (Çamcı et al.), event-related potentials (ERP) (Nanova), and magnetoencephalography (MEG) (Potts et al.). All adopt original approaches, investigating uncommon and innovative objects of study, such as pareidolia, mental rotation, and motor imagery or “visualization,” while characterizing the underlying mental processes as a theoretical foundation for interpreting behavioral, ERP, and MEG measures.

Çamcı et al. investigate gender attribution to ambiguous faces (face pareidolia) in patients receiving emergency medical care. Specifically, a substantial 81% of the pareidolia faces were categorized as male, and this finding was consistent across both male and female patients. Thus, male patients were more likely to perceive ambiguous faces as congruent with their own gender, whereas female patients showed the opposite pattern, with the dominant reference being incongruent with their own gender. Intriguingly, while attributions of male gender dominated in frequency, patients were significantly faster at attributing a female gender (1.44 s) to ambiguous pareidolia faces than a male gender (3.25 s). This discrepancy invites further investigation of how automatic visual processing interacts with social and cultural expectations in gender attribution in pareidolia.

A narrative review led by Nanova examine**s** sex-related differences in mental rotation in young adults. Synthesizing event-related potential (ERP) evidence across studies, the review highlights that sex differences in mental rotation, particularly during demanding behavioral 3D tasks, are accompanied by differences in ERP amplitude, latency, global field power, and scalp topography. Nanova further notes that neurophysiological differences may occur even when behavioral performance is comparable, suggesting that accuracy and response time alone may not fully capture sex-related differences in the neurocognitive processes underlying mental rotation.

Potts et al. further examine motor imagery in elite female ice hockey players using exclusively non-behavioral measures in a MEG study. The authors identified, even at the individual level, a principal hub of neural activity during the imagery condition in a posterior left hemisphere cortical region surrounding the intraparietal sulcus. This initiative calls for further studies that expand the neurocognitive interpretation of MEG results, advancing the characterization of mental processes that occur without equivalent behavioral measures, such as response option or execution time. Gender-specific aspects regarding motor imagery, taking into consideration cultural aspects and previous experience, would also be further investigated.

Together, these female-led studies clearly embody the dual mission of this Research Topic: they elevate the visibility of women leading rigorous neuroscientific investigation while simultaneously advancing how sex and gender are conceptualized, measured, and interpreted in modern neuroscience.

By amplifying women's perspectives, this Research Topic contributes to a more equitable and broadly informative social neuroscience.
